# Supplementary figures and images for: Diagnosis of invasive pulmonary aspergillosis using metagenomic next-generation sequencing and conventional microbial tests post-COVID-19 pandemic
Source: Microbiol Spectr. 2025 Jun 9;13(7):e00121-25. doi: 10.1128/spectrum.00121-25 (PMC12210878; doi:10.1128/spectrum.00121-25)

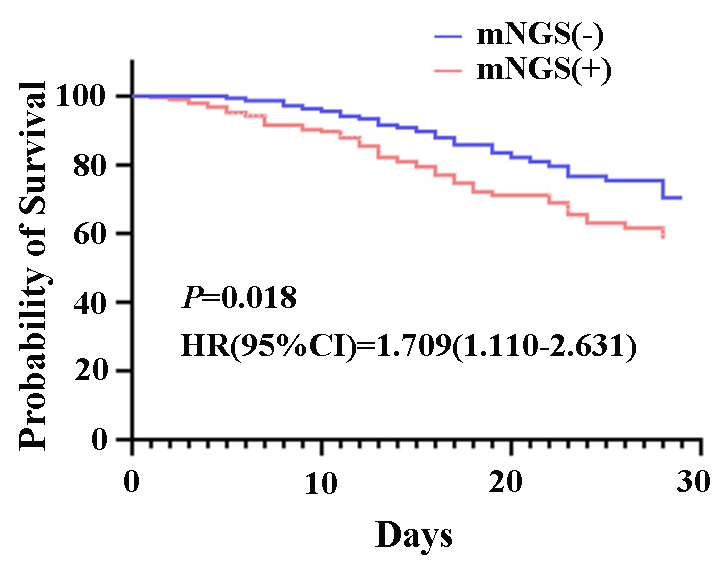

Supplement: Figure S1 — Overall survival analysis for mNGS (+) and mNGS (-) patients. [file spectrum.00121-25-s0001.tif]

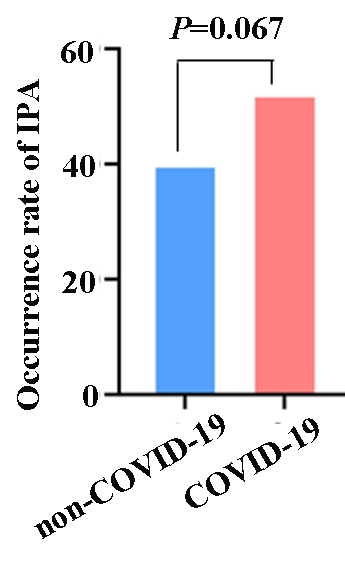

Supplement: Figure S2 — The incidence of IPA in COVID-19 and non-COVID-19 groups. [file spectrum.00121-25-s0002.tif]

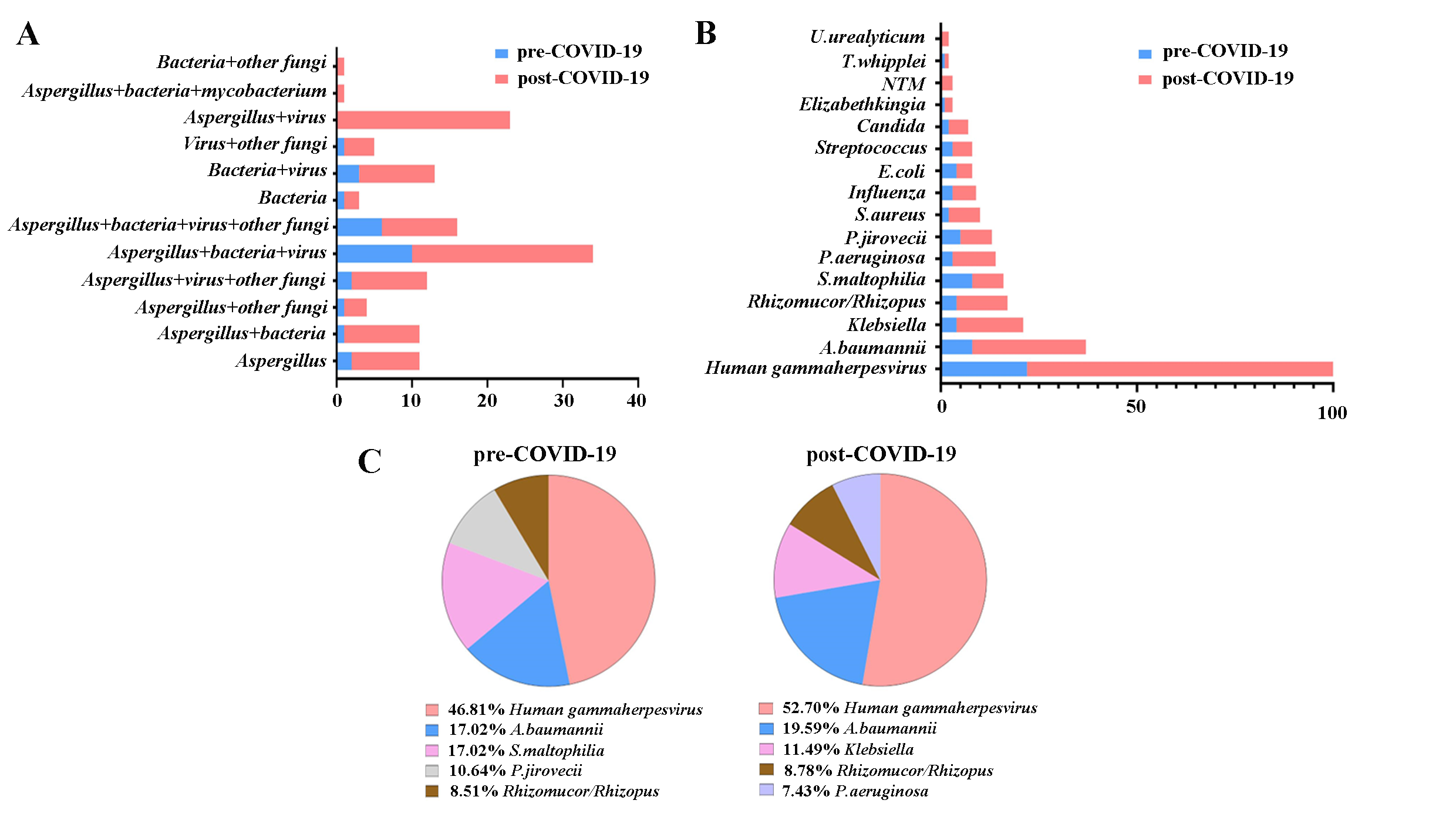

Supplement: Figure S3 — Mixed infections and the predominant pathogens identified in IPA patients before and postCOVID-19 pandemic. [file spectrum.00121-25-s0003.tif]
