# Supplementary material for: Diagnosis of invasive pulmonary aspergillosis using metagenomic next-generation sequencing and conventional microbial tests post-COVID-19 pandemic
Source: Microbiol Spectr. 2025 Jun 9;13(7):e00121-25. doi: 10.1128/spectrum.00121-25 (PMC12210878; doi:10.1128/spectrum.00121-25)
Supplement: Supplemental material — Additional experimental details. [file spectrum.00121-25-s0004.docx]

**Appendix 1.**

**Figure S legend**

Figure S1: Overall survival analysis for mNGS (+) and mNGS (-) patients

Figure S2: The incidence of IPA in COVID-19 and non-COVID-19 groups.

Figure S3: Mixed infections and the predominant pathogens identified in IPA patients before and post COVID-19 pandemic.

A. The number of cases with different infection patterns in patients affected by COVID-19. B. The number of cases with different pathogens identified in patients affected by COVID-19. C. The top five predominant pathogens identified in pre-COVID-19 and post-COVID-19 patients, respectively.

**Appendix 2.**

**2.1 Procedures of Culture**

About 5 ml of bronchoalveolar lavage fluid (BALF) sample was centrifuged at 2,000 × g for 10 minutes to concentrate fungal elements. Then, we use the sediment resuspended in 1-2 mL sterile saline for inoculation. Finally, 0.5 mL of processed BALF was inoculated onto Blood agar and Sabouraud dextrose agar, spreading evenly, and incubated at 35℃ for 2-3 days in aerobic conditions.

**2.2 Procedures of Smear Microscopy**

First, we centrifuged 5 ml of BALF at 2,000 × g for 10 minutes, discarded the supernatant, and thoroughly mixed the sediment. Then, we took a drop and evenly spread it on a clean slide, added one drop of fluorescent staining solution, covered with a coverslip, and waited for 1 minute before conducting microscopic examination.

**2.3 Procedures of GM tests**

We conducted the GM test using Platelia™ *Aspergillus* Ag (Bio-Rad, USA) in accordance with the manufacturer's reagent instructions. Briefly, serum samples were centrifuged at 10,000 × g for 10 minutes at room temperature to remove particulate matter. For BALF, samples were centrifuged at 3,000 × g for 10 minutes, and the supernatant was aliquoted into sterile microcentrifuge tubes. The Platelia™ *Aspergillus* Ag kit components (microplate strips, conjugate, controls, sample diluent, and substrate) were equilibrated to room temperature (18-25°C) for 30 minutes. All the samples were then processed with plate configuration, sample and conjugate addition, washing, substrate addition, and the final reaction termination and measurement at 450 nm.

**2.4 Detailed Procedures of Metagenomic Next-generation Sequencing**

**2.4.1 Sample Acquisition, Processing, and DNA Extraction**

Samples of 5 mL bronchoalveolar lavage fluid (BALF) were collected from patients according to standard procedures, and were immediately sent to the clinical laboratory. Briefly, 1 mL of sample was centrifuged at 12,000 × g for 5 min to collect the pathogens and human cells. Next, 50 μL of precipitate underwent depletion of host nucleic acid using 1 U of Benzonase (Sigma) and 0.5% Tween 20 (Sigma) and incubated at 37°C for 5 min^(1)^. Terminal buffer (400 μL) was added to stop the reaction. Then, the quantified unique DNA fragments (named UMSI) were spiked for each sample as an identity and internal control, which were PCR products of Oryza sativa 400 to 600 bp in length. A total of 600 μL of the mixture was transferred to new tubes containing 500 μL of ceramic beads for bead beating using a Minilys personal TGrinder H24 homogenizer (catalog number OSE-TH-01; Tiangen, China). Then, the nucleic acid from 400 μL of pretreated samples was extracted and eluted in 60 μL of elution buffer using a QIAamp UCP pathogen minikit (catalog number 50214; Qiagen, Germany). The extracted DNA was quantified using a Qubit double-stranded DNA (dsDNA) high-sensitivity (HS) assay kit (catalog number Q32854; Invitrogen, USA).

**2.4.2 Construction of DNA library**

Fragmentation and tagmentation of the DNA were performed using the bead-linked transposome. After completion of posttagmentation cleanup, the tagmented DNA was amplified; the thermocycling parameters were as follows: 72℃ for 3 min and 98°C for 30 s, followed by 17 cycles of 15 s at 98°C, 30 s at 60°C, and 30 s at 72°C, before 5 minutes at 72°C. Purification and size selection were carried out following the double-sided bead purification procedure. A Qubit dsDNA HS assay kit was used to measure the library concentration with an Invitrogen™ Qubit™ 4 Fluorometer. The library was prepared by pooling a 1.5 pM concentration of each purified sample equally for sequencing on an MGISEQ 200 sequencer using a 50-cycle single-end sequencing strategy.

**2.4.3 Sequencing and Bioinformatic Analyses**

For bioinformatics analysis, Trimmomatic was used to remove low-quality reads, adapter contamination and duplicate reads^(2)^. Low-complexity reads were removed by Kcomplexity using default parameters. The human sequence data were identified and excluded by mapping to a human reference genome (hg38) using SNAP v1.0beta.18^(3)^. To construct the microbial genome database, pathogens and their genomes or assemblies were selected following the Kraken2 criteria for selecting representative assemblies for microorganisms (bacteria, viruses, fungi, protozoa, and other multicellular eukaryotic pathogens) from the NCBI Assembly and Genome databases (https://benlangmead.github.io/aws-indexes/k2). Microbial reads were aligned to the database using Burrows-Wheeler Aligner software^(4)^. We defined that reads with 90% identity of reference were mapped reads. In addition, reads with multiple locus alignments within the same genus were excluded from the secondary analysis. Only reads mapped to the genome within the same species were considered.

**2.4.4 Interpretation of metagenomic data and an official report to the clinician**

We normalized the sequencing reads RPTM to eliminate the errors caused by various sequencing depths among samples. To establish the optimal threshold value for the >10 microbes with culture isolates, samples spiked with microbes were defined as positive samples, while negative control (NC) was defined as the negative sample. Receiver operating characteristic curves were plotted for each target species using these samples. The parameter resulting in the highest area AUC was considered the positive cutoff value for this species ^(5)^. For microorganisms without culture isolates, the RPTM mean value and standard deviation of this microorganism were calculated, and the RPTM (mean + 2 standard deviations [SD]) was set as a positive cutoff value ^(6)^.

The clinical reportable range (CRR) for pathogens was established according to the following three references indicated in a previous study^(6)^: (i) the Johns Hopkins ABX Guide (https://www.hopkinsguides.com/hopkins/index/Johns_Hopkins_ABX_Guide/Pathogens), (ii) Manual of Clinical Microbiology^(7)^, and (iii) clinical case reports or research articles published in peer-reviewed journals. All microbes that exceeded the threshold of mNGS were classified into 3 categories^(8)^: (i) probable (BALF mNGS-based results were within the CRR and concordant with the clinical and radiologic results; the RPTM was significantly higher than the positive cutoff value, and the abundance was obviously higher than that of other species of the same genus), (ii) possible (the microbe has pathogenic potential, but an alternate explanation is more likely), and (iii) unlikely (the microbe cannot cause pneumonia).

To monitor the sources of potential contamination, both NC and sterile deionized water, which served as nontemplate controls, were prepared in parallel with other samples in each batch^(9)^. In addition, we used sterile cotton swabs dipped in sterile deionized water to wipe the surfaces of the centrifuge and biosafety cabinet to generate the background microorganism list in our laboratory.

**Reference**

1. World Health Organization. 2020. WHO reveals leading causes of death and disability worldwide: 2000-2019.<https://www.who.int/news/item/>09-12-2020-who-reveals-leading-causes-of-death-and- disability - worldwide-2000-2019. Accessed 9 December 2020.

2. Bolger AM, Lohse M, Usadel B. 2014. Trimmomatic: a flexible trimmer for Illumina sequence data. Bioinformatics 30:2114–2120. doi:10.1093/bioinformatics/btu170.

3. Zaharia M, Bolosky WJ, Curtis K, Fox A, Patterson D, Shenker S, Stoica I, Karp RM, Sittler T. 2011. Faster and more accurate sequence alignment with SNAP. arXiv 1111.5572 [cs.DS]. https://arxiv.org/abs/1111.5572.

4. Li H, Durbin R. 2009. Fast and accurate short read alignment with Burrows-Wheeler transform. Bioinformatics 25:1754–1760.doi: 10.1093/bioinformatics/btp324.

5. Unal I. 2017. Defining an optimal cut-point value in ROC analysis: an alternative approach. Comput Math Methods Med 2017:3762651. doi: 10.1155/2017/3762651.

6. Jing C, Chen H, Liang Y, Zhong Y, Wang Q, Li L, Sun S, Guo Y, Wang R, Jiang Z, Wang H. 2021. Clinical evaluation of an improved metagenomic next-generation sequencing test for the diagnosis of bloodstream infections. Clin Chem 67:1133–1143. doi: 10.1093/clinchem/hvab061.

7. Jorgensen JH, Carroll KC, Funke G, Pfaller MA, Landry ML, Richter SS, Warnock DW (ed). 2015. Manual of clinical microbiology, 11th ed. ASM Press, Washington, DC.

8. Chen H, Yin Y, Gao H, Guo Y, Dong Z, Wang X, Zhang Y, Yang S, Peng Q, Liu Y, Wang H. 2020. Clinical utility of in-house metagenomic next-generation sequencing for the diagnosis of lower respiratory tract infections and analysis of the host immune response. Clin Infect Dis 71:416–426.

9. Miller S, Naccache SN, Samayoa E, Messacar K, Arevalo S, Federman S, Stryke D, Pham E, Fung B, Bolosky WJ, Ingebrigtsen D, Lorizio W, Paff SM, Leake JA, Pesano R, DeBiasi R, Dominguez S, Chiu CY. 2019. Laboratory validation of a clinical metagenomic sequencing assay for pathogen detection in cerebrospinal fluid. Genome Res 29:831–842. doi: 10.1101/gr.238170.118.
